# Performance evaluation of alternative bacteriological measures of response to MDR-TB therapy during the initial 16 weeks of treatment

**DOI:** 10.21203/rs.3.rs-5834681/v1

**Published:** 2025-04-10

**Authors:** Willy Ssengooba, Emmanuel Musisi, Derrick Semugenze, Kevin Komakech, Moses Ndema, Christine Wiltshire Sekaggya, Susan Adakun, Derek J Sloan, Achilles Katamba, Mohammed Lamorde, Moses Joloba, Wilber Sabiiti

**Affiliations:** Department of Medical Microbiology, and Makerere University Biomedical Research Center (MAKBRC), College of Health Sciences Makerere University; Division of Infection and Global Health, School of Medicine, University of St Andrews, KY16 9TF St Andrews.; Department of Medical Microbiology, and Makerere University Biomedical Research Center (MAKBRC), College of Health Sciences Makerere University; Department of Medical Microbiology, and Makerere University Biomedical Research Center (MAKBRC), College of Health Sciences Makerere University; Adult Tuberculosis Unit, Mulago National Referral and Teaching Hospital; Infectious Diseases Institute, Makerere University College of Health Sciences; Adult Tuberculosis Unit, Mulago National Referral and Teaching Hospital; Division of Infection and Global Health, School of Medicine, University of St Andrews, KY16 9TF St Andrews.; Makerere University Lung Institute; Infectious Diseases Institute, Makerere University College of Health Sciences; Department of Medical Microbiology, and Makerere University Biomedical Research Center (MAKBRC), College of Health Sciences Makerere University; Division of Infection and Global Health, School of Medicine, University of St Andrews, KY16 9TF St Andrews.

## Abstract

**Background:**

Monitoring response to Multi-Drug-Resistant Tuberculosis (MDR-TB) treatment is burdensome to TB programmes and may benefit from alternative effective tools. We evaluated the concordance of alternative bacteriological measures of response to therapy (AMRT) during the initial sixteen weeks of MDR-TB treatment.

**Methods:**

In a prospective study of MDR/RR-TB among smear positive adults, aged 18 year and above. Pooled early morning- and spot sputa were obtained before treatment initiation (95% on Bdq, Lzd, Lfx, Cfz, Cs regimen) and at weeks 2, 4, 6, 8, 12, and 16 during treatment between 14/02/2020 and 09/02/2024. Samples were tested using Concentrated Fluorescent Microscopy (CFM), Fluorescein-diacetate (FDA)-Acid Fast Bacilli (AFB) vital smear microscopy, the tuberculosis-Molecular bacterial load assay (TB-MBLA), and Middle brook 7H11 selective (MB7H11S) colony-forming units as the AMRT. Concordance of the AMRT for sputum conversion was compared to Mycobacterial Growth Indicator Tube (MGIT) culture conversion at weeks 12 and 16 of treatment.

**Results:**

A total of 101 MDR/RR-TB patients were screened of which 42 were smear negative. Fifty-nine participants were enrolled, of whom 58 (98%) provided baseline sputa and these were included in the analysis. The concordance, n/N (%) of each AMRT test with MGIT culture conversion at week 12 were: 31/35(88.6%) for CFM, 32/33 (97.0%) for FDA, and 25/26 (96.2%) for TB-MBLA, and 11/11 (100%) for MB7H11S. At week 16, concordance of eachAMRT were: 39/40 (97.5%) for CFM, 35/36 (97.2%) for FDA, 32/32 (100%) for TB-MBLA, and 15/15 (100%) for MB7H11S. Among people living with HIV,the concordances of AMRT with MGIT culture conversion varied at week 8 but was 100% for all tests at weeks 12 and 16. Baseline clinical and/or bacteriological factors did not influence the concordance of AMRT to MGIT culture conversion at weeks 12, and 16.

**Conclusion:**

Our data show that concentrated Fluorescent smear, Fluorescein-di-acetate smear microscopy, and TB-MBLA are suitable alternative measures of response to TB therapy compared to MGIT culture among MDR-TB participants. Use of these alternative rapid methods may allow timely decision making as well as rapid evaluation of alternative MDR-TB treatment regimens.

## BACKGROUND

Tuberculosis (TB) remains a major global challenge despite the availability of effective TB treatment regimen for more than 50 years [1]. Treating Multidrug resistant TB (MDR-TB) disease remains challenged by long and complicated treatment regimens coupled with suboptimal treatment outcomes [1]. There is an increasing risk of morbidity and mortality associated with MD-RTB with associated increase in transmission [1]. The rates of treatment success for both drug susceptible (DS) and Drug Resistant (DR) TB participants remains low[1]. There is therefore an urgent need to improve treatment success rates. Consequently, routine treatment monitoring and shortening time to treatment decisions is crucially beneficial to MDR-TB patients.

The on-going spread of MDR-TB, defined as resistance to rifampicin and isoniazid, is threatening TB control efforts [2]. In 2023, Uganda reported the prevalence of MDR-TB as 1.1% and 3% among new and previously treated TB individuals respectively[1]. MDR-TB is difficult to treat and cure, requiring lengthy treatment with multiple toxic drugs. The World Health Organization (WHO) suggests the use of the 9-month all-oral regimen rather than longer (18-month) regimens in patients with MDR/RR-TB and in whom resistance to fluoroquinolones has been excluded and a 6-month treatment regimen composed of bedaquiline, pretomanid, linezolid (600 mg) and moxifloxacin (BPaLM) under specific conditions[3].

There is a huge need for evaluating new drug combinations containing novel drugs such as the bedaquiline containing regimens to simplify and further shorten the treatment period[4]. The interval and sensitivity of treatment monitoring method is vital to document early therapeutic failure which may impact particularly the implementation of the new BPAL and BPALM regimens [5]. This calls for rapid and low-cost treatment response monitoring methods for most high burden low- and middle-income countries LMICs).

Conventional sputum smear microscopy is the most common TB test in resource limited settings but it is unsuitable for measuring treatment response as it does not differentiate between dead and live bacilli in the smear. Fluorescein di-acetate (FDA) vital stain microscopy test detects live bacteria in smear and may expedite diagnosis of poor response to treatment, predict treatment failure and relapse [6, 7].

The GeneXpert MTB/RIF (Xpert) has been a game changer for rapid detection of rifampicin resistance with increased sensitivity observed in a newer version of cartridge the Xpert^®^ MTB/RIF Ultra (Ultra; Cepheid, Sunnyvale, CA, USA) assay. The Xpert MTB/RIF test detectsDNA that persists long after cell death and this limits it from being a suitable treatment response monitoring tool [8]. The Tuberculosis Molecular bacterial load assay (TB-MBLA) is a quantitative polymerase chain reaction (RT-qPCR) test that quantifies changes in *M. tuberculosis* rRNA during treatment [9]. This culture-free biomarker is rapid and accurate among DS-TB patients [9-11]. TB-MBLA performance data among MDR/RR-TB is not readily available. The related bacterial phenotypic changes usually delays time to culture conversion for MDR/RR-TB compared to drug susceptible TB[12].

Middlebrook 7H11 agar has been used to measure *M. tuberculosis* colony-forming units per mL (cfu/mL)[13]. A more sensitive Mycobacterial Growth Indicator Tube (MGIT) culture, a liquid culture, is the gold standard method for measuring response to TB treatment. This method is sensitive; however, it is prone to contamination, requires specialized laboratory infrastructure, highly skilled personnel and difficult to decentralize. In this study we evaluated the concordance of the alternative bacteriological measures of response to therapy (AMRT) compared to MGIT culture among MDR/RR-TB patients during the first 16-weeks of treatment.

## MATERIALS AND METHODS

### Study design, and site

This prospective observational study was conducted at Mulago National Referral Hospital- Kampala, Uganda between 14/02/2020 and 09/02/2024. Participants who were found to have drug resistant TB were admitted to MDR-TB ward and managed for 8-weeks according to the national policy by then[14]. After discharge from the hospital, participants were followed up monthly for clinical examination, drug refills and study related data collection up to the end of treatment [13].

### Study participants

Study participants were consenting adult male and female individuals, aged 18-years and above and had positive test result for drug resistant TB on either GeneXpert MTB/RIF/ULTRA and/or line probe assay (LPA). Participants were MDR-TB treatment naïve with productive cough, residing in greater Kampala region, and with ability to return to the testing facility during the treatment follow-up phase.

### TB treatment and follow up

Patients were initiated on a regimen containing combinational medicines including:Bedaquiline (Bdq), Ethambutol (E), Cycloserine (Cs), Linezolid (Lzd), Clofazimine (Cfz), Ethionamide (Eto), Pyrazinamide (Z), Izoniazid (H), Moxifloxacin (Mfx). At the end of 16 weeks of active treatment follow-up phase, participants were passively followed-up at 9- and 18-months using phone calls to document their treatment outcomes and to rule-out relapse. The WHO specified TB treatment outcome definition was used [15].

### Sample collection procedures.

**Figure 1:**
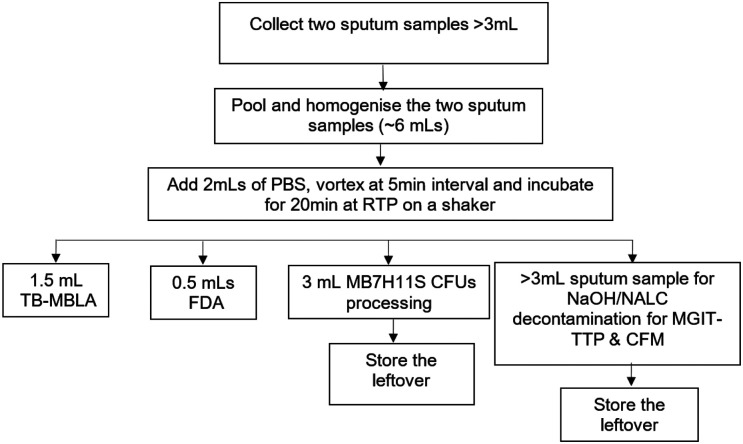
Sample management for laboratory procedures

Expectorated early morning and spot sputa were collected, pooled, homogenized and portioned before testing at week 0 (before treatment initiation), and at every 2, 4, 6, 8, 10, 12 and 16 weeks of treatment, Fig. 1. During the initial 8 weeks, sputum collection was observed and supervised by the study nurse or a laboratory technician in the hospital’s designated sputum collection area. After discharge from the hospital- during the continuation phase, participants self-collected an overnight- and additional spot samples at every visit point but with guiding instructions from the study nurse. Samples and the accompanying requisition forms were referred to the Mycobacteriology laboratory (BSL-3) for analysis. This facility is in the Department of Medical Microbiology, College of Health Sciences, Makerere University Kampala-Uganda and it is accredited by the College of American Pathologists (CAP:ISO15189).

Figure 1 summarizes the sample preparation and portioning for specific alternative bacteriological measures of response to MDR-TB treatment.

### Laboratory procedures

#### Tuberculosis Molecular Bacteria Load Assay (TB-MBLA):

The portion for TB-MBLA was preserved by adding 4ml of guanidine thiocyanate (GTC) and stored until batch testing. Total *M. tuberculosis* rRNA was extracted using chloroform-phenol method and then tested at 0.1 dilution. TB-MBLA test was performed based on the duplex reverse transcriptase-real time qPCR principle targeting both *M. tuberculosis complex* and the extraction control using a RotorGene 5plex platform (Qiagen, Manchester, UK). PCR cycling conditions were as reported by Honeyborne, *et. al*[11]. Quantification cycle (Cq) readouts were converted to bacterial load using a standard curve that was customized for the site's qPCR platform and recorded as estimated colony forming units per mL (eCFU/mL). Samples without Cq values, and those with Cq values above 30.5 were reported as TB negative [10]. A portion of the extracted RNA was stored in the H3-Africa biorepository on site for future studies.

### Middlebrook 7H11 Selective (MB7H11S)

Middlebrook 7H11S was made inhouse by adding 25 μg/mL of carbenicillin, 5 μg/mL of amphotericin B, 10 μg/mL of trimethoprim and 100 units/mL of polymixin B during media preparation. Raw sputum was homogenized with 10% sputazol solution and a10-fold dilutions of it prepared in Saline-Tween 80. Culture plates were inoculated with 100 μL of each dilution in duplicates, sealed with carbon dioxide-permeable tape and placed at 37°C in a carbon dioxide (5–10%) incubator. Plates were examined for contamination at day 3 and for growth from week 1 to week 8. Visible colonies were enumerated each time the plates were read till week 8. Colony forming units per mill (CFU/mL) were calculated by multiplying the average number of colonies by the dilution factor. H37Rv laboratory strain of 0.5 McFarland was used as a positive control.

### Fluorescein diacetate (FDA) vital staining microscopy

Two smears were prepared from the most mucoid part of unprocessed sputum and air-dried in a biosafety cabinet for at least 1 hour. Filter papers were placed in petri dishes, humidified with sterile distilled water and the non-fixed slides placed on support sticks in the petri dish. The slides were flooded with 0.25 mg/ml FDA solution per slide and incubated at 34-38°C for 30 minutes. They were washed and decolorized with 0.5% acid alcohol for 2 minutes, counter stained with 0.5% potassium permanganate for 1 minute and flooded with 5% phenol solution to kill the bacilli for 10 minutes. Slides were air-dried away from direct sunlight and examined immediately using fluorescent microscope. They were graded as presence or absence of AFB using the WHO/IUATLD scale at 200x magnification.

### Sputum decontamination

Early morning and spot sputum samples were pooled and homogenized. Each mL of the homogenized sputum was decontaminated usingNaOH/N-acetyl L-cysteine (NALC) (i.e., fresh 2% solution prepared with 2.9% trisodium citrate and 0.5 g NALC). The resultant was centrifuged for 15 minutes, and the supernatant was decanted to recover a pellet, which was neutralized in 2 mL of sterile phosphate-buffered saline (PBS; pH 6·8; Becton Dickinson, Sparks, MD, USA).

#### Mycobacteria Growth Indicator Tube (MGIT):

MGIT tubes were inoculated with 500 μL of the decontaminated sputum sample and incubated at 37°C for a maximum of 42 days. MTB-positive cultures were confirmed by the presence of acid-fast bacilli on Ziehl–Neelsen staining and the presence of MPT64 antigen. Absence of acid-fast bacilli cording, and growth on blood agar was recorded as contamination. All results were reported according to the standard procedures[16].

### Concentrated Fluorescent smear microscopy

Following specimen decontamination with N-acetyl-L-cysteine–sodium citrate–NaOH method, and inoculating the MGIT culture, 100 μl (2 drops) of well-mixed resuspended pellet was spread on a pre-labelled frosted end slide over an area of approximately 1 x 2 cm. Slides were air-dried and heat-fixed on a slide warmer at a temperature between 65°C to 75°C for at least 2 hours. Dried slides were stained using auramine O method. Briefly, 1% auramine O stain was flooded on the smear for 20 minutes, washed and decolorized with 0.5% acid alcohol for 2 minutes before counter staining with 0.5% potassium permanganate for 1 minute. Slides were air-dried away from direct sunlight and examined immediately using fluorescent microscope. They were graded as presence or absence of AFB using the WHO/IUATLD scale at 200x magnification.

#### Statistical analysis:

Differences in baseline continuous variables including, quantification cycles, and TB-MBLA-measured bacterial loads were compared using Mann-Whitney U-test. The concordance of AMRT sputum conversion compared with MGIT culture conversion during MDR-TB treatment at weeks 12 and 16 was calculated. These concordances were compared among HIV positive participants. Factors influencing the concordance of the alternative measures of response to MDR-TB treatment compared with MGIT culture forweek 12-, and 16- sputum culture conversion as well as favorable treatment outcome were analyzed in a logistic regression model. Factors including baseline smear grade, drug susceptibility results, being HIV positive, history of previous TB treatment, being on ART, history of smoking and alcohol use were considered to influence the concordance of alternative measures of response with MGIT culture conversion. Factors having a P-value less than 0.2 in a bivariate model were included in a multivariate model. Factors with P-value less than 0.05 at 95% confidence interval (CI) were considered statistically significant.

### Ethical consideration

The study was approved by the Makerere University School of Biomedical Sciences Research Ethics committee (SBS-REC #651) and the Uganda National Council for Science and Technology (UNCST #HS471ES)

## RESULTS

### Baseline clinical Characteristics

A total of 101 MDR/RR-TB patients were screened of which 42 were smear negative. Fifty-nine participants were enrolled, of whom 58 (98%) provided baseline sputa and these were included in the analysis. Participants were mainly young adults with median (IQR) age 33 years (28.6–37.4). Out of the 58 participants, 37 (63.8%) were males, 25 (43.9%) were living with HIV, and 32 (55.2%) reported a history of previously treated TB. We observed that 29/45 (64.4%) were resistant to both rifampicin and isoniazid, and that 20/55 (36.4%) had abnormal baseline chest X-ray. Majority 18/25(72.0%) of those living with HIV were on antiretroviral therapy by the time of enrolment. More than half 35 (61.4%) of the participants were underweight with BMI < 18.5kg/m^2,^ Table 1.

### Baseline laboratory characteristics

Although all participants were smear positive on enrollement, baseline tests had different sensitivities. Positivity rates n (%) were higher for MGIT (51 (98.1%) compared to 49 (84.5%) for CFM, and 47(81%) for Middle Brook 7H11 Selective (MB7H11S). Positivity rates n (%) were 40 (69.0%) and 32 (60.4%) for FDA smear microscopy and TB-MBLA, respectively. Baseline resistance profiles varied and majority of the participants, 29/58 (64.4%) were resistant to isoniazid and majority of the patients, 55 (94.8) were initiated on the Bdq, Lzd, Lfx, Cfz, Cs regimen (see Table 2).

### Changes in positivity rates

**Figure 2:**
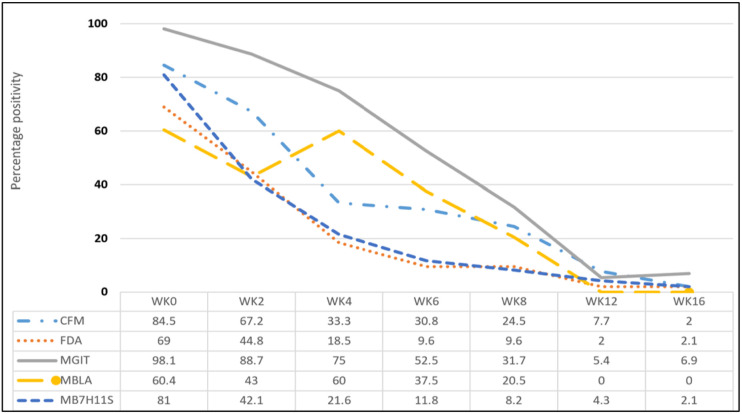
Percentage changes in bacteriological positivity per week by test method

**Figure 3:**
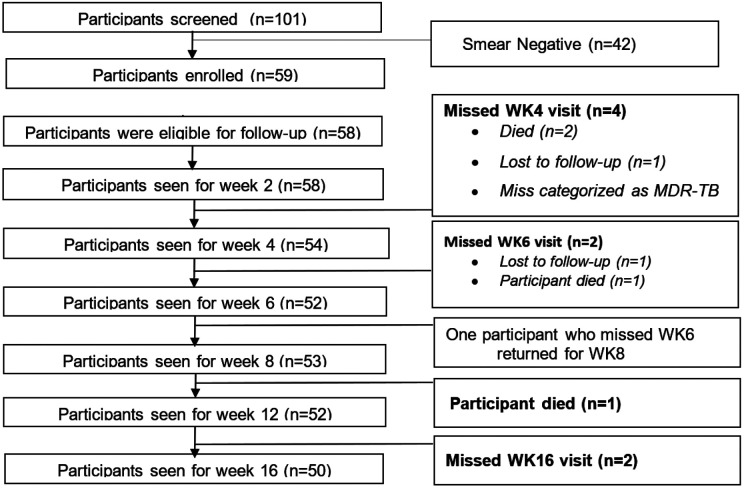
Recruitment and follow-up flow chart

Generally, majority (above 85%) of the participants were retained in the study during the treatment follow up phase. Treatment outcomes were as follows: 6 (10.3%) completed treatment, 43 (74.1%) were declared cured, 7 (12.1%) died and 2 (3.4%) was lost to follow-up at the end of treatment. Positivity rates significantly reduced across the treatment monitoring methods by weeks 12 and 16, Fig. 2. Participants were followed up until week 16 and a total of 50/58 (86.2%) were retained in the study, Fig. 3. The percentage positivity by the methods used were CFM 4/52 (7.7%) and 1/50 (2.0%), FDA smear microscopy were 1/50 (2.0%) and 1/46 (2.2%), TBMBLA 1/40 (2.5%) and 0/40 (0%), MGIT 2/37(5.4%) and 3/43 (7.0%) and MB7H11S 2/47 (4.3%) and 1/47 (2.1%) for week 12 and 16 respectively, Table 3.

#### Concordance of different alternative bacteriological measures of response to therapy at week 12 and 16 using MGIT culture conversion as a reference comparator.

The concordance, n/N (%) of each AMRT test at week 12 with MGIT culture conversion were as follows: 31/35(88.6%) for CFM, 32/33 (97.0%) FDA, and 25/26 (96.2%) TB-MBLA and it was 11/11 (100%) for MB7H11S. At week 16 were: 39/40 (97.5%) CFM, 35/36 (97.2%) for FDA, and 32/32 (100%) TB-MBLA and it was 15/15 (100%) for MB7H11S, Table 4. Among the people living with HIV, the concordances for culture conversion varied at week 8 but was 100% for all tests at weeks 12 and 16, Table 5.

#### Baseline bacteriological and patient characteristics associated with weeks 12, and 16 culture conversion.

Baseline smear grade, drug susceptibility results, being HIV positive, history of previous TB treatment, being on ART, history of smoking and alcohol were not statistically associated with AMRT MGIT culture conversion at weeks 12, and 16.

## DISCUSION

In this prospective study of MDR/RR-TB participants during the initial 16 weeks of treatment, we demonstrate the concordance of alternative bacteriological measures of response to MDR-TB therapy that is consistent with WHO target product profile for triage and confirmatory diagnostic tests[17].

Specifically, we found that CFM and FDA vital staining smear microscopy, TBMBLA and middlebrook 7H11 selective are suitable alternative measures of response to therapy among MDR/RR-TB patients compared to MGIT cultures. More than 90% of the participant who culture converted were also negative by the alternative methods by weeks 12 and 16. Furthermore, the participants who converted by alternative bacteriological measures also had favourable treatment out comes with no relapse. The concordance of AMRT with MGIT culture conversion at weeks 12 and 16 was not different by HIV status.

Monthly cultures for treatment monitoring are recommended by the WHO[3, 18], however, culture is less accessible, requires specialized laboratories and skill and takes long to yield results. It is important to note that these alternative bacteriological methods are accessible and could potentially support MDR/RR-TB patient management. MGIT culture remains less accessible due to high operational cost, high skills demand, longer turnaround time and contamination. This calls for a rapid and low-cost methods for most high burden low-income countries. Smear microscopy, the most used method in resource limited settings, remains less specific for measuring treatment response as it does not differentiate between dead and live bacilli. Fluorescein di-acetate (FDA) vital stain microscopy has been reported to detect live bacteria in smear. Treatment monitoring using FDA method has been found to expedite diagnosis of poor response to treatment as well as quantifying early response to treatment as the change in percentage raise in percentage lipid body in a positive AFB smear over the first four weeks may predict failure/relapse[6]. A study with fewer patients indicated that a change in FDA and quantitative culture results during early treatment differed significantly between patients with non-MDR tuberculosis and those with MDR tuberculosis [7]. Furthermore, Researchers at the University of St Andrews, UK evaluated the tuberculosis molecular bacterial load assay (TB-MBLA) as a fast and accurate means for monitoring tuberculosis treatment response among 92% drug susceptible tuberculosis participants in which bacterial load correlated to the rise in MGIT-TTP (p<0.001 spearman’s correlation rank test). TB-MBLA measures *M. tuberculosis* 16S ribosomal RNA using a more affordable kit, less infrastructural requirements and results available within 4 hours. And TB-MBLA standard operating procedures (SOP) have been published in 2017[9, 10]. Based on operational data, the TB-MBLA test is easy to perform with minimal training and the cost per test of $22 is comparable to unsubsidized Xpert and far lower than that of culture. Plans to automate TB-MBLA are under way, a company called Lifearc in Edinburgh has been engaged to start the process. TB-MBLA is a potential game-changer for treatment monitoring especially among MDR-TB participants to protect the already limited treatment options[19-21].

The standard method to measure the efficacy of a drug or treatment regimen as well as phase II clinical trials for novel drugs is through early bactericidal activity (EBA) studies which show reduction of *M. tuberculosis* burden in patient’s sputum over 14 days of treatment. EBA studies require high skills and operational costs and cannot be used to follow individual participants’ treatment response on an MDR-TB regimen. Time to culture conversion for MDR/RR-TB is usually longer than that of drug susceptible TB [7]. Even regular monthly cultures, as recommended by the WHO for follow-up of the treatment response among MDR/RR-TB patients[3], are difficult since culture laboratories are frequently unavailable. Cultures have high rates of contamination, required highly skilled personnel, difficult to decentralize, have high safety requirements, and negative cultures take several months. These risks introducing high costs associated with long delays in bringing novel drugs to market during the development cycle especially for phase II clinical trials as well as delayed treatment decision making during MDR-TB treatment. MDR-TB patients treated with effective second-line treatment generally converts to culture negative after a median of three months treatment [22, 23]. Culture using Middlebrook 7H11 solid media is cheaper in terms of supplies, equipment and infrastructural requirements and may be an alternative to MGIT culture.

Few clinical studies follow up participants long enough to identify predictors of poor MDR-TB treatment outcome. We followed up participants longer than previous studies and beyond the expected median conversion time, to the end of the intensive phase. This enabled us to document the ability of alternative bacteriological measures to detect long-term converters/persisters. Moreover, 75% of the participants in this study were reported as cured and 10% as completed treatment. This gave a treatment success rate of 84% which is comparable to the 85% registered by Uganda in 2022. Moreover, MDR-TB treatment failure detection has been found to depend on monitoring interval and microbiological method [5].

Low body weight, long duration of illness, cavitary disease and alcohol and tobacco use have been found to influence outcome of MDR-TB participants on treatment [24]. On contrary, our study among others, we found none of these influencing culture conversion or MDR-TB treatment success[25]. Several studies have evaluated these alternative methods among drug susceptible TB patients with a few among DR-TB patients[7, 21, 26]. Our study is one of the few studies evaluated the alternative methods head-to-head for treatment response monitoring, with a long follow-up period, among MDR/RR-TB patients.

## Conclusion

In our study, we have demonstrated that concentrated fluorescent and fluorescein-di-acetate smear microscopy, TBMBLA and middlebrook7H11 selective as suitable alternative measures of response to therapy among MDR-TB participants compared to Mycobacterial Growth indicator tube (MGIT) culture. These alternative measures of response to MDR-TB treatment are cheap and more accessible compared to MGIT culture. Using these methods will enable the National TB Control Programs (NTPs) to have better estimates of treatment outcomes, most especially the cure outcome which is usually under-reported for most MDR-TB participants completing treatment.

## Figures and Tables

**Table 1:** Baseline characteristics of participants

Characteristic	Frequency (%)
**Gender**	
Male	37 (63.8)
Female	21 (36.2)
Age, years, median (IQR)	33 (28.6–37.4)
**HIV status**	
Positive	25 (43.1)
Negative	32 (55.2)
Unknown	1(1.7)
**On ART**	
Yes	18 (72.0)
No	7 (28.0)
**Underweight (BMI < 18.5kg/m^2^)**	
Yes	35 (61.4)
No	22 (38.6)
Median BMI kg/m^2^	18.1 (17.3–18.6)
**Previously diagnosed with TB**	
Yes	32 (55.2)
No	26 (44.8)
**Marital status**	
Single	18 (31.0)
Married	20 (34.5)
Separated	17 (29.3)
Widowed	3 (5.2)
**Education level**	
None	5 (8.6)
Incomplete primary	15 (25.9)
**Gender**	
Completed Primary	12 (20.7)
Incomplete Secondary	12 (20.7)
Completed Secondary	7 (12.1)
Tertiary	7 (12.1)
**Household member diagnosed with TB in the last year**	
Yes	11 (19.0)
No	47 (81.0)
**History of smoking**	
Yes	21 (36.2)
No	36 (62.1)
Unknown	1 (1.7)
**History of Alcohol use**	
Yes	25 (43.1)
No	33 (56.9)
**History of diabetes**	
Yes	6 (10.3)
No	52 (89.7)
**History of cancer**	
Yes	1 (1.7)
No	57 (98.3)
**Any information about TB**	
Yes	38 (65.5)
No	20 (34.5)
**Family history of TB**	
Yes	15 (25.9)
No	26 (44.8)
Unknown	1 (1.7)
**Gender**	
**Care sought before this visit**	
Yes	54 (93.1)
No	4 (6.9)
**Given medication?**	
Yes	39 (70.9)
No	16 (29.1)
**Fever**	
Yes	45 (77.6)
No	13 (22.4)
**Weight loss**	
Yes	52 (89.7)
No	6 (10.3)
**Night sweats**	
Yes	50 (86.2)
No	8 (13.8)
**Chest pain**	
Yes	42 (72.4)
No	16 (27.6)
**Chest X-ray**	
Normal	35 (63.6)
Abnormal	20 (36.4)

**Table 2:** Baseline clinical and laboratory characteristics of participants

Test	n (%)
Laboratory positivity	
CFM	49/58 (84.5)
FDA	40/58 (69.0)
TBMBLA	32/53 (60.4)
Median (IQR) MBLA Ct value	24.70 (20.86–26.51)
MGIT	51/52 (98.1)
Median (IQR) MGIT/TTP days/hour	5.18 (5.0–6.13)
MB7H11S	47/58 (81.0)
Median (IQR) MB7H11S/log CFU/mL	2.63 (2.36–2.81)
Drug Resistance	
Rifampicin	58 (100)
Isoniazid (n = 45)	29 (64.4)
Ethambutol (n = 45)	8 (17.8)
Pyrazinamide (n = 33)	3 (9.1)
Bedaquiline (n = 43)	1 (2.3)
Linezolid (n = 27)	1 (3.7)
Levofloxacin (n = 47)	2 (4.3)
Moxifloxacin (n = 43)	1 (2.3)
Treatment Regimen	
Bdq, E, Cs, Lzd, Cfz	1 (1.7)
Bdq, Lfx, Cfz, Cs, Eto, Z	1 (1.7)
Bdq, Lzd, Lfx, Cfz, Cs	55 (94.8)
Eto, E, H, Mfx, Cfz	1(1.7)

CFM= Concentrated Fluorescent Microscopy, FDA= Fluorescein Diacetate, TBMBLA= Tuberculosis Molecular Bacterial Load Assay, TTP= Time To Positivity, MGIT= Mycobacterial Growth Indicator Tube, MB7H11S= Middle Brook 7H11 Selective, CFU= Colony Forming Units, IQR= Interquartile Range, Ct= Cycle threshold, Bdq=Bedaquiline, E= Ethambutol, Cs= Cycloserine, Lzd = Linezolid, Cfz= clofazimine, Eto= Ethionamide, Z= Pyrazinamide, H= Izoniazid, Mfx= Moxifloxacin

**Table 3:** Percentage of bacteriological positivity per week by test method

Test/Week	Week 0	Week 2	Week 4	Week 6	Week 8	Week 12	Week 16
CFM	49/58 (84.5%)	39/58 (67.2%)	18/54 (33.3%)	16/52 (30.8%)	13/53 (24.5%)	4/52 (7.7%)	1/50 (2.0%)
CFM Median grade	2 (2–3)	2 (2–3)	2.5 (1–3)	2 (1–2)	2 (2–2)*	2 (1–2)*	4 (4–4)*
FDA	40/58 (69.0%)	26/58 (45.0%)	10/54 (18.5%)	5/52 (9.6%)	5/52 (9.6%)	1/50 (2.0%)	1/46 (2.2%)
FDA median (IQR) grade	2(1.7-2)	2 (2–3)	2 (2–3)	2 (1.3-2)	1 (1–2)*	1 (1–1)*	4 (4–4)*
TBMBLA	32/53 (60.4%)	24/55 (43.6%)	29/50 (58.0%)	18/48 (37.5%)	9/44 (20.4%)	1/40 (2.5%)	-----
Median (IQR) MBLA Ct value	24.71 (20.8-26.51)	24.04 (20.98–25.74)	26.51 (23.84–27.38)	26.07 (23.76–28.07)	26.41 (23.67–29.72)	------	-----
MGIT	51/52 (98.1%)	47/53 (88.7%)	36/48 (75.0%)	21/40 (52.5%)	13/41 (31.7%)	2/37 (5.4%)	3/43 (6.98%)
Median (IQR) MGIT/TTP days/hrs	5.18 (5.0-6.13)	11.18 (10.04–13.18)	13.18 (12.29–17.36)	15.2 (10.57–21.63)	16.07 (10.95–24.14)	12.01 (3.06–13.07)*	9.07 (1.05–10.12)*
MGIT contamination rate	6/58 (10.3)	5/58 (8.6)	6/54 (11.1)	12/52 (23.1)	12/53 (22.6)	15/52 (28.8)	7/50 (14.0)
MB7H11S	47/58 (81.0%)	24/57 (42.1%)	11/51 (21.6%)	6/51 (11.76)	4/49 (8.2%)	2/47 (4.3%)	1/47 (2.1%)
Median (IQR) MB7H11S/log CFU/mL	2.63 (2.36–2.81)	2.31 (1.94–2.70)	2.07 (1.34–2.72)	1.30 (1.0-0.77)*	2.63 (1.90–2.62)*	1.74 (1.69–1.77)*	2.04 (1.04–2.04)*

* Lower (upper) confidence limit held at minimum (maximum) of the sample, Binary data are n/N (%), Quantitative data are median (IQR), smear grade score; Actual number=1, 1+=2, 2+=3, 3+=4, CFM= Concentrated Fluorescent Microscopy, FDA= Fluorescein Diacetate, TBMBLA= Tuberculosis Molecular Bacterial Load Assay, TTP = Time To Positivity, MGIT= Mycobacterial Growth Indicator Tube, MB7H11S= Middle Brook 7H11 Selective, CFU= Colony Forming Units, IQR= Interquartile Range, Ct= Cycle threshold

**Table 4:** Concordance of the alternative measures of response to MDR-TB treatment compared to MGIT culture

Method	CFM concordance n/N (%)		FDA Concordance n/N (%)		TBMBLA Concordance n/N (%)		MB7H11S concordance n/N (%)	
Weeks	Pos	Neg	Pos	Neg	Pos	Neg	Pos	Neg
**W2**	33/47 **(70.2)**	4/6 **(66.7)**	23/47 **(48.9)**	5/6 **(83.3)**	19/43 **(44.2)**	4/6 **(66.9)**	7/19 **(36.8)**	5/5 **(100)**
**W4**	16/36 **(44.4)**	12/12 **(100)**	8/36 **(22.2)**	12/12 **(100)**	22/33 **(66.7)**	6/8 **(75.0)**	4/11 **(36.4)**	7/7 **(100)**
**W6**	10/21 **(47.6)**	16/19 **(84.2)**	5/21 **(23.8)**	19/19 **(100)**	10/21 **(47.6)**	16/19 **(84.2)**	1/9 **(11.1)**	9/9 **(100)**
**W8**	8/13 **(61.5)**	26/28 **(92.9)**	3/13 **(23.1)**	26/27 **(96.3)**	1/7 **(14.3)**	18/22 **(81.8)**	1/4 **(25.0)**	11/11 **(100)**
**W12**	0/2 **(0.0)**	31/35 **(88.6)**	0/2 **(0.0)**	32/33 **97.0)**	0/2 **(0.0)**	25/26 **(96.2)**	1/1 **(100)**	11/11 **(100)**
**W16**	0/3 **(0.0)**	39/40 **(97.5)**	0/3 **(0.0)**	35/36 **(97.2)**	----*	32/32 **(100)**	---*	15/15 **(100)**

-------*: All negative by the test and no false positive, CFM=Concentrated Fluorescent Microscopy, FDA=, TB MBLA– Tuberculosis Molecular Bacterial Load Assay, MGIT= Mycobacterial Growth Indicator Tube, MB7H11S= Middle Brook 7H11 Selective, W=Weeks, Pos = positive, Neg=Negative

**Table 5:** Concordance of the alternative measures of response to MDR-TB treatment compared to MGIT among HIV-Positive participants

Method	CFM concordance n/N (%)		FDA concordance n/N (%)		TBMBLA concordance n/N (%)		MB7H11S concordance n/N (%)	
Weeks	Pos	Neg	Pos	Neg	Pos	Neg	Pos	Neg
**W2**	14/19 **(73.7)**	4/5 **(100)**	10/19 **(52.6)**	5/5 **(100)**	8/17 **(47.1)**	4/5 **(80.0)**	7/19 **(36.8)**	5/5 **(100)**
**W4**	8/14 **(38.1)**	7/7 **(100)**	4/14 **(28.6)**	7/7 **(100)**	8/13 **(61.5)**	3/4 **(75.0)**	4/11 **(36.4)**	7/7 **(100)**
**W6**	4/9 **(44.4)**	9/9 **(100)**	2/9 **(22.2)**	9/9 **(100)**	3/7 **(42.9)**	6/9 **(66.7)**	1/9 **(11.1)**	9/9 **(100)**
**W8**	2/4 **(50.0)**	12/13 **(92.3)**	2/4 **(50.0)**	12/13 **(92.3)**	0/2 **(0.0)**	7/11 **(63.6)**	1/4 **(25.0)**	11/11 **(100)**
**W12**	-----*	15/15 **(100)**	-----*	14/14 **(100)**	----*	11/11 **(100)**	1/1 **(100)**	11/11 **(100)**
**W16**	-----*	17/17 **(100)**	----*	14/14 **(100)**	---*	13/13 **(100)**	-----*	15/15 **(100)**

------*: All negative by the test and no false positive, CFM=Concentrated Fluorescent Microscopy, FDA=, TB MBLA– Tuberculosis Molecular Bacterial Load Assay, MGIT= Mycobacterial Growth Indicator Tube, MB7H11S= Middle Brook 7H11 Selective, W=Weeks, Pos = positive, Neg=Negative
